# Gap junctions are selectively associated with interlocking ball-and-sockets but not protrusions in the lens

**Published:** 2010-11-09

**Authors:** Sondip K. Biswas, Jai Eun Lee, Lawrence Brako, Jean X. Jiang, Woo-Kuen Lo

**Affiliations:** 1 Department of Neurobiology, Morehouse School of Medicine, Atlanta, GA; 2 Department of Ophthalmology, Emory University, Atlanta, GA; 3 Department of Biochemistry, University of Texas Health Science Center at San Antonio, TX

## Abstract

**Purpose:**

Ball-and-sockets and protrusions are specialized interlocking membrane domains between lens fibers of all species studied. Ball-and-sockets and protrusions are similar in their shape, size, and surface morphology, and are traditionally believed to play a key role in maintaining fiber-to-fiber stability. Here, we evaluate the hypothesis that ball-and-sockets and protrusions possess important structural and functional differences during fiber cell differentiation and maturation.

**Methods:**

Intact lenses of leghorn chickens (E7 days to P62 weeks old) and rhesus monkeys (1.5–20 years old) were studied with SEM, freeze-fracture TEM, freeze-fracture immunogold labeling (FRIL), and filipin cytochemistry for membrane cholesterol detection.

**Results:**

SEM showed that ball-and-sockets were distributed along the long and short sides of hexagonal fiber cells, whereas protrusions were located along the cell corners, from superficial to deep cortical regions in both chicken and monkey lenses. Importantly, by freeze-fracture TEM, we discovered the selective association of gap junctions with all ball-and-sockets examined, but not with protrusions, in both species. In the embryonic chicken lens (E18), the abundant distribution of ball-and-socket gap junctions was regularly found in an approximate zone extending at least 300 μm deep from the equatorial surface of the superficial cortical fibers. Many ball-and-socket gap junctions often protruded deeply into neighboring cells. However, in the mature fibers of monkey lenses, several ball-and-sockets exhibited only partial occupancy of gap junctions with disorganized connexons, possibly due to degradation of gap junctions during fiber maturation and aging. FRIL analysis confirmed that both connexin46 (Cx46) and connexin50 (Cx50) antibodies specifically labeled ball-and-socket gap junctions, but not protrusions. Furthermore, filipin cytochemistry revealed that the ball-and-socket gap junctions contained different amounts of cholesterol (i.e., cholesterol-rich versus cholesterol-free) as seen with the filipin-cholesterol-complexes (FCC) in different cortical regions during maturation. In contrast, the protrusions contained consistently high cholesterol amounts (i.e., 402 FCCs/μm^2^ membrane) which were approximately two times greater than that of the cholesterol-rich gap junctions (i.e., 188 FCCs/μm^2^ membrane) found in ball-and-sockets.

**Conclusions:**

Gap junctions are regularly associated with all ball-and-sockets examined in metabolically active young cortical fibers, but not with protrusions, in both chicken and monkey lenses. Since these unique gap junctions often protrude deeply into neighboring cells to increase membrane surface areas, they may significantly facilitate cell-to-cell communication between young cortical fiber cells. In particular, the large number of ball-and-socket gap junctions found near the equatorial region may effectively facilitate the flow of outward current toward the equatorial surface for internal circulation of ions in the lens. In contrast, a consistent distribution of high concentrations of cholesterol in protrusions would make the protrusion membrane less deformable and would be more suitable for maintaining fiber-to-fiber stability during visual accommodation. Thus, the ball-and-sockets and protrusions are two structurally and functionally distinct membrane domains in the lens.

## Introduction

The lens is composed of numerous sheets of slender fiber cells covered by a monolayer of epithelium at its anterior surface. The lens permits incident light to pass through and help form a focused image on the retina through the mechanism of accommodation. The lens possesses several unique features that serve these specific functions. It contains a high concentration of crystallin proteins which increase the refractive index. The lens has no blood supply and its mature fiber cells lose their organelles during the maturation process to eliminate light scattering. Metabolic activities in the lens are low and proceed anaerobically, and the exchanges of ions and small metabolites between lens cells depend on gap junctions [1-4].

Gap junctions between lens fiber cells of various species have been demonstrated by morphological, physiologic and biochemical studies [1,2,5-13]. Fiber gap junctions exhibit unique structural characteristics and distribution in cortical fibers. For example, the typical 2–4 nm intercellular gap has rarely been seen in the fiber gap junctions of the intact lens [2,9,10,14-17]. Also, gap junctions are distributed mainly in a single row along the middle of the narrow sides of hexagonal fiber cells, but have a random distribution on their wide sides [8,18]. In addition, the presence of gap junctions in the interlocking ball-and-sockets of cortical fibers has been reported sporadically in several species studied [2,10,16,19]. Recently, Biswas et al. [20,21] have revealed that gap junctions contain different amounts of cholesterol and undergo structural remodeling during fiber cell differentiation and maturation in the chicken lens. Biochemical and molecular studies have identified two gap junction proteins (connexins [Cx]) in the lens fibers of various species such as the mouse (Cx46 and Cx50) [22,23], sheep (Cx49 and Cx44) [24-26], bovine (Cx44 and Cx50) [27], and chicken (Cx45.6 and Cx56) [28,29]. The chicks Cx50 (formerly Cx45.6) and Cx46 (formerly Cx56) are used in this study to follow the nomenclature of human and rodent homologs to avoid confusion.

In addition, lens fibers possess an elaborate interlocking system [14,30-36] and adherens junctions [37-43] for maintaining their structural order and stability which are critical requirements for lens transparency, especially during the deformation which accompanies visual accommodation. Interlocking connections between lens fibers exhibit many different configurations and are basically in the forms of ball-and-sockets, protrusions and tongue-and-grooved ridges in various species studied [14,31-33,35,44-46].

Large numbers of ball-and-sockets are regularly found in the superficial cortex and, at reduced frequency, in the deeper cortex in many species examined. They are distributed most abundantly along the long sides of the hexagonal fiber cells [31-33,35]. Structurally, the ball-and-sockets typically consist of the head and neck portions with head diameters in the range of 0.5–2 μm.

Numerous protrusions resemble ball-and-socket domains but are located primarily at the corners of hexagonal cortical fiber cells [31-33,35]. The term `protrusion’ is used in this study according to the classification by Willekens et al. [32,33,35], although other terms, such as interdigitations, spikes and interlocking devices have been used for the same structure [5,14,31,47]. Like ball-and-sockets, the protrusions also contain the head and neck portions with head diameters in the range of 0.1–1.5 µm in many species [31-33,35,36]. However, unlike ball-and-sockets, protrusions are distributed more extensively in the mature cortical fibers and nuclear fibers of the lens [32-36]. Because ball-and-sockets and protrusions are similar in shape, size and surface morphology, they have not been clearly separated in several lens studies [5,14,18,30,47]. They are both traditionally believed to play a key role in maintaining fiber-to-fiber stability, important for normal visual accommodation.

In this study, we evaluate the hypothesis that the interlocking ball-and-sockets and protrusions possess important structural and functional differences during fiber cell differentiation and maturation. We systematically analyze these two types of interlocking devices in chicken and monkey lenses, using scanning and thin-section TEM, freeze-fracture TEM, filipin cytochemistry, and freeze-fracture replica immunogold labeling. Our results show that despite the similarities in shape, size and surface morphology, gap junctions are selectively associated with all ball-and-sockets examined (i.e., 142 for chicken lenses and 103 for monkey lenses) but not with protrusions in both species. Freeze-fracture immunogold labeling confirms that both Cx46 and Cx50 antibodies are specifically localized in the ball-and-socket gap junctions, but not in the protrusions, in chicken lenses. Filipin cytochemistry further demonstrates that there are different cholesterol distributions in the ball-and-socket gap junctions during fiber cell differentiation and maturation. In contrast, the protrusions in various cortical regions consistently contain high cholesterol amounts which are approximately two times higher than that of the cholesterol-rich gap junctions found in ball-and-sockets. This study suggests that the unique ball-and-socket gap junctions may significantly facilitate their communicating role between metabolically active young fiber cells by protruding deeply into the neighboring cells to increase membrane surface areas for cell-to-cell communication. In particular, the large number of ball-and-socket gap junctions found near the equatorial region may effectively facilitate the flow of outward current toward the equatorial surface for internal circulation of ions in the lens. In contrast, the presence of consistently high cholesterol content in the protrusions would make these domains less deformable, and would be more suitable for maintaining fiber-to-fiber stability. This study demonstrates that ball-and-sockets and protrusions are two structurally and functionally distinct membrane domains in the lens.

## Methods

### Animals

Fertile white leghorn chicken eggs (Hyline International, Mansfield, GA) were incubated at 38 °C in a humidified incubator (Petersime, Gettysburg, OH) for 7–20 days. After the appropriate incubation period, the embryonic lenses were then surgically isolated. Adult white leghorn chickens (42–62 weeks old) were purchased from a local poultry farm (Hyline International). Freshly isolated eyeballs of rhesus monkeys (1.5 years to 20 years old), collected in ice-cold PBS solution, were kindly provided by the Yerkes National Primate Research Center of Emory University, Atlanta, GA. All lenses were removed from freshly enucleated eyeballs and fixed immediately in the fixative for the various experiments described below. The animals were treated in accordance with the Association for Research in Vision and Ophthalmology Resolution on the Use of Animals in Research.

### Scanning electron microscopy

Freshly isolated lenses of chickens and monkeys at various ages were fixed in 2.5% glutaraldehyde in 0.1 M cacodylate buffer, pH 7.3 at room temperature for 48–72 h. Each lens was properly orientated and fractured with a needle or sharp razor blade to expose the short or long sides of the membrane surfaces of hexagonal fiber cells. Lens halves were then post-fixed in 1% aqueous OsO_4_ for 1–2 h at room temperature, dehydrated in graded ethanol and dried in a Samdri-795 critical point dryer (Tousimis Inc., Rockville, MD). Lens halves were oriented and mounted on specimen stubs and coated with gold/palladium in a Hummer VII sputter coater (Anatech Inc., Union City, CA). Some lens halves of monkeys were fractured for coating after critical point drying to obtain better membrane surfaces of the long-side fiber cells. Micrographs were taken with a JEOL 820 scanning electron microscope (JEOL, Peabody, MA) at 10 kV.

### Thin-section electron microscopy

All freshly isolated lenses of embryonic chickens at various ages were fixed in an improved fixative containing 2.5% glutaraldehyde, 0.1 M cacodylate buffer (pH 7.3), 50 mM L-lysine and 1% tannic acid for 2 h at room temperature [37]. Each lens was then mounted on a specimen holder with superglue and cut into 200 µm slices with a Vibratome. Each lens was carefully oriented on the specimen holder such that either a cross- or longitudinal section of cortical fibers could be obtained initially with a Vibratome. The lens nucleus in older lenses often separated from its cortex after sectioning. Thus, only doughnut-shaped lens slices of cortical fibers could be collected in 0.1 M cacodylate buffer for this study. Lens slices were then post-fixed in 1% aqueous OsO_4_ for 1 h at room temperature, rinsed in distilled water and stained en bloc with 0.5% uranyl acetate in 0.15 M NaCl overnight at 4 °C. Tissue slices were dehydrated through graded ethanol and propylene oxide, and embedded in Polybed 812 resin (Polysciences, Warrington, PA). Thick sections (1 µm) cut with a glass knife were stained with 1% toluidine blue and examined with a light microscope to select the area of interest. Thin sections (80 nm) were cut with a diamond knife, stained with 5% uranyl acetate followed by Reynold's lead citrate and examined in a JEOL 1200EX electron microscope (JEOL).

### Freeze-fracture TEM and cytochemical detection of membrane cholesterol

Freshly isolated lenses of chickens and monkeys were fixed in 2.5% glutaraldehyde in 0.1M cacodylate buffer (pH 7.3) at RT for 2–4 h. After washing in buffer, lenses were orientated to obtain sagittal (longitudinal) sections with a Vibratome, and slices were collected, marked serially from superficial to deep and kept separately. Because the dimension of each Vibratome slice of the adult chicken lens was too large for routine freeze fracturing, the areas of interest were carefully dissected out into small rectangular or square blocks (~2×2 mm) from the anterior-central or posterior-central surface of lens slices. These slices were then cryoprotected with 25% glycerol in 0.1 M cacodylate buffer at RT for 1 h and processed for freeze-fracture TEM according to our routine procedures [20]. In brief, a single lens slice was mounted on a gold specimen carrier and frozen rapidly in liquefied Freon 22 and stored in liquid nitrogen. Cryofractures of frozen slices were made in a modified Balzers 400T freeze-fracture unit (Boeckeler Instruments, Inc., Tucson, AZ), at a stage temperature of −135 °C in a vacuum of approximately 2 × 10^−7^ Torr. The lens tissue was fractured by scraping a steel knife across its frozen surface to expose fiber cell membranes. The fractured surface was then immediately replicated with platinum (~2 nm thick) followed by carbon film (~25 nm thick). The replicas, obtained by unidirectional shadowing, were cleaned with household bleach and examined with the transmission electron microscope.

For cytochemical detection of filipin-cholesterol-complexes (FCCs) with freeze-fracture TEM, we followed the identical procedures as described previously [20,21]. Due to the lack of monkey lens tissue, filipin cytochemical experiments were conducted only for the chicken lenses.

### Freeze-fracture replica immunogold labeling (FRIL)

Freshly isolated chicken lenses were lightly fixed in 0.75% paraformaldehyde in PBS for 30–45 min at RT, and then cut into 300 μm slices with a Vibratome to make freeze-fracture replicas. One drop of 0.5% parloidion in amyl acetate was used to secure the integrity of the whole piece of a large replica during cleaning and immunogold labeling procedures. The replica was washed with 2.5% sodium dodecyl sulfate, 10 mM Tris-HCl, 30mM sucrose, pH 8.3 (SDS buffer) at 50 °C until all visible attached tissue debris was removed from the replica. The replica was then rinsed with PBS, blocked with 4% BSA-0.5% teleostean gelatin in PBS for 30 min and incubated with the affinity purified rabbit anti-Cx50 and anti-Cx46 polyclonal antibodies made from the COOH-terminus [48] at 1:10 dilution for 1 h at RT. The replica was washed with PBS and incubated with 10 nm Protein A gold (EY Laboratories, San Mateo, CA) at 1:50 dilution for 1 h at RT. After rinsing, the replica was fixed in 0.5% glutaraldehyde in PBS for 10 min, rinsed in water, collected on a 200-mesh Gilder finder grid, rinsed with 100% amyl acetate for 30 s to remove parloidion and viewed with a JEOL 1200EX TEM.

## Results

### Distribution of ball-and-socket domains in the chicken and monkey lenses

**Figure 1:**
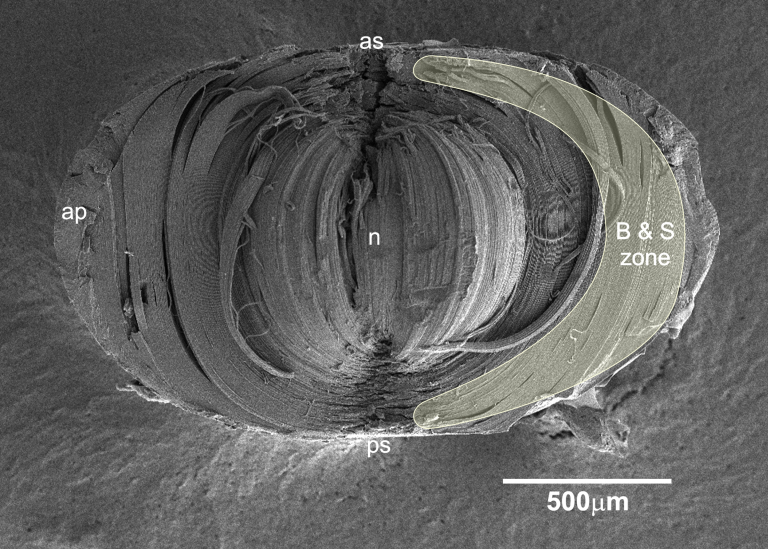
SEM overview of fiber cell membrane surfaces of fractured intact embryonic chicken lens (E18) along the anterior/posterior (median sagittal) axis. This fractured axis is for examination of ball-and-sockets on the short sides of cortical fiber cells. An approximate zone for the abundant distribution of ball-and-sockets is outlined. In the equatorial region, the ball-and-socket zone extends at least 300 μm deep from the surface. In the images, B&S zone, ball-and-socket zone; AP, annular pad; N, nucleus; AS, anterior surface; PS, posterior surface.

**Figure 2:**
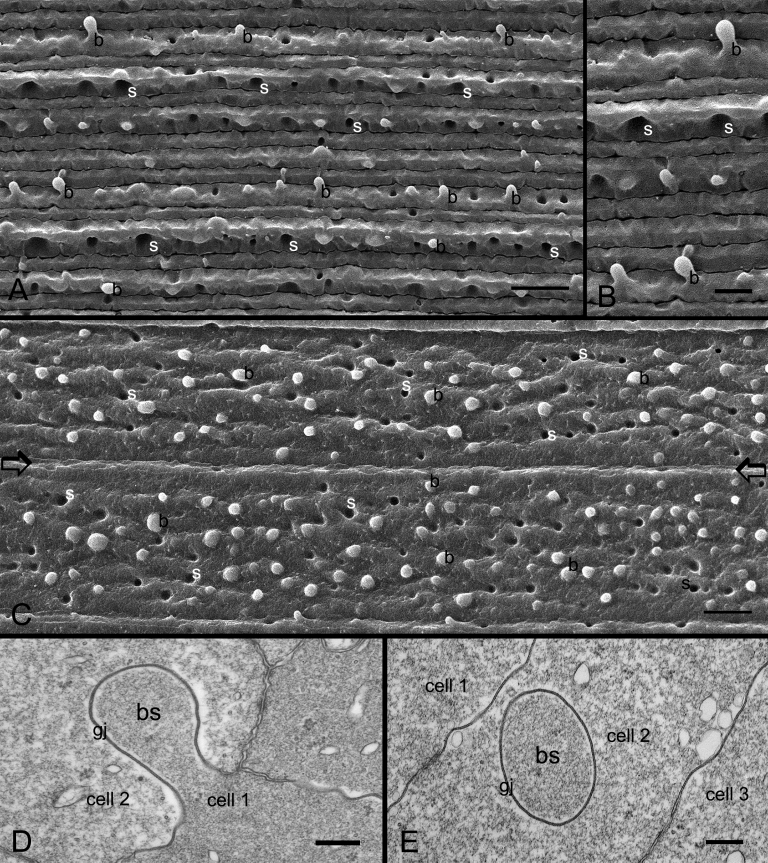
SEM and thin-section TEM of ball-and-sockets in the embryonic and adult chicken lenses. **A**: In the embryonic chicken lens (E18), a representative scanning electron micrograph showing many ball-and-sockets distributed on the short sides of superficial cortical fibers at the equatorial region (approximately 100 μm from the surface). **B**: At higher magnification, the height and shape of the ball-and-sockets can be readily visualized from side-view. **C**: In the adult chicken lens (P42 weeks), numerous ball-and-sockets are seen distributed in rows on the two long sides of superficial cortical fibers at the equatorial region (approximately 200 μm from the surface). It is estimated that the number of ball-and-sockets (approximately 13 ball-and-sockets per 100 μm^2^ membrane) is not significantly different at the apical, equatorial and posterior regions along the anterior-posterior axis of any given superficial fiber cell. The border between two long-side fiber cells is marked by two arrows. Note that the height and the shape of these top-viewed ball-and-sockets cannot be readily appreciated as compared with those seen from the short sides in **A** and **B**. **D**: Thin-section TEM shows a ball-and-socket with the head and neck portions protruding into an adjacent cell. This ball-and-socket is completely occupied by a gap junction (gj). **E**: A cross-section of the ball-and-socket gap junction showing a considerable membrane extension of this junction into a narrow fiber cell. In the images, b is the ball, and s is the socket. The scale bar indicates 5 μm in **A**, **C**, 2 μm in **B**, and 200 nm in **D** and **E**.

By scanning electron microscopy, fiber-cell membrane surfaces of intact chicken lens (E18), fractured along the anterior/posterior (median sagittal) axis, were used to examine the distribution of ball-and-sockets on the short sides of cortical fiber cells (Figure 1). The abundant distribution of ball-and-sockets was regularly found in an approximate zone extending at least 300 μm deep from the equatorial surface of the superficial cortical fibers (Figure 1). A representative SEM micrograph shows that many ball-and-sockets were regularly distributed along the short sides of superficial cortical fibers at the equatorial region (approximately 100 μm from the surface) in the E18 embryonic chicken lens (Figure 2A). We were able to clearly visualize the heights and distinct shapes of the head and neck of ball-and-socket domains from this good orientation of the short-side fiber cells (Figure 2B). When the ball-and-sockets were examined on the long sides of superficial cortical fibers (approximately 200 μm from the equatorial surface), many of them were seen randomly distributed along the entire width (approximately 10 μm) of the long sides of the fiber cells in the adult chicken lens (Figure 2C). It was estimated that the number of ball-and-sockets was approximately 13 ball-and-sockets per 100 μm^2^ membrane and was not significantly different at the apical, equatorial and posterior regions along the anterior-posterior axis of any given fiber cell.

**Figure 3:**
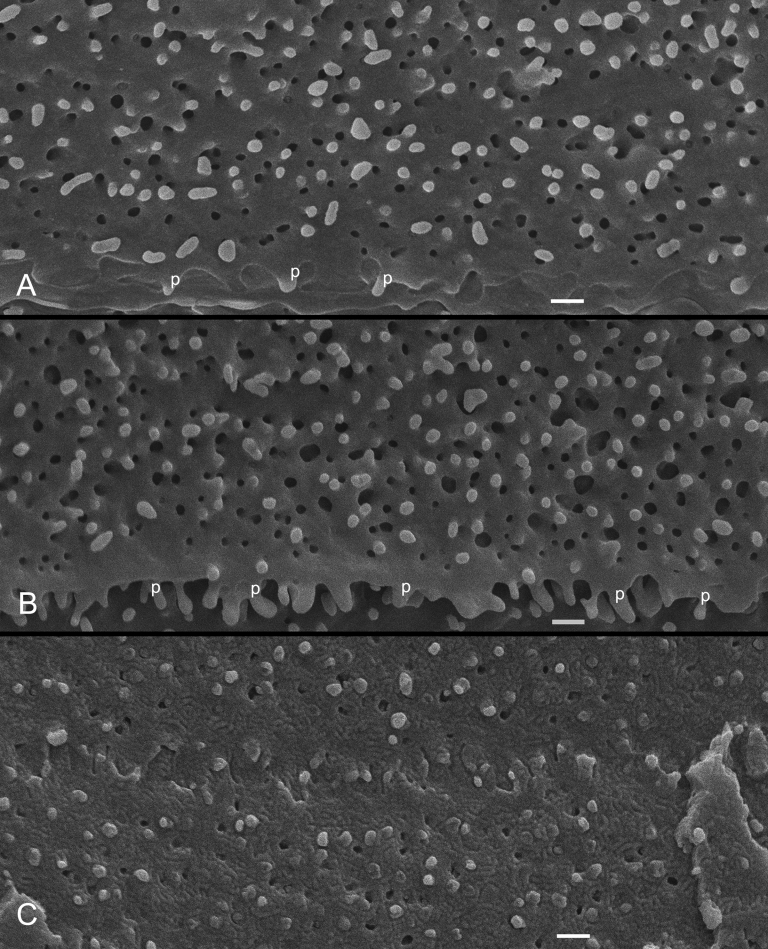
SEM of ball-and-sockets in different cortical regions of monkey lens (20 year old). **A**: Superficial cortical fibers (approximately 100 μm from the surface), numerous ball-and-sockets are distributed on the long side of fiber cells. **B**: Intermediate cortical fibers (approximately 300 μm from the surface), a large number of ball-and-sockets are found on the long side of fiber cells. In this region, many protrusions (p) are also distributed along the corners of cortical fiber cells. **C**: However, in the deeper cortex (approximately 500 μm from the surface), ball-and-sockets display smaller number and size with degenerating appearance. The scale bars indicate 1 μm.

In the young and adult monkey lenses examined (e.g., 1.5–20 years old), ball-and-sockets were distributed in large amounts on the long sides of cortical fiber cells of various regions along the anterior/posterior axis of the lens. At the equatorial region, they were found in superficial young cortical fibers at approximately 100 μm from the surface (Figure 3A), mid-cortical fibers at approximately 300 μm from the surface (Figure 3B), and deeper mature cortical fibers at approximately 500 μm from the surface (Figure 3C). The number and the size of ball-and-sockets were reduced in the deeper mature cortical fibers, and many of these ball-and-sockets exhibited degenerating appearance (Figure 3C).

### Gap junctions were present in all ball-and-sockets examined in both chicken and monkey lenses

By thin-section TEM, the specific association of gap junctions with ball-and-sockets in fiber cells of the chicken lens was consistently revealed (Figure 2D,E). Both the longitudinal (Figure 2D) and cross-sectional (Figure 2E) views of ball-and-sockets all demonstrated that the ball-and-socket gap junctions protruded deeply into neighboring cells.

**Figure 4:**
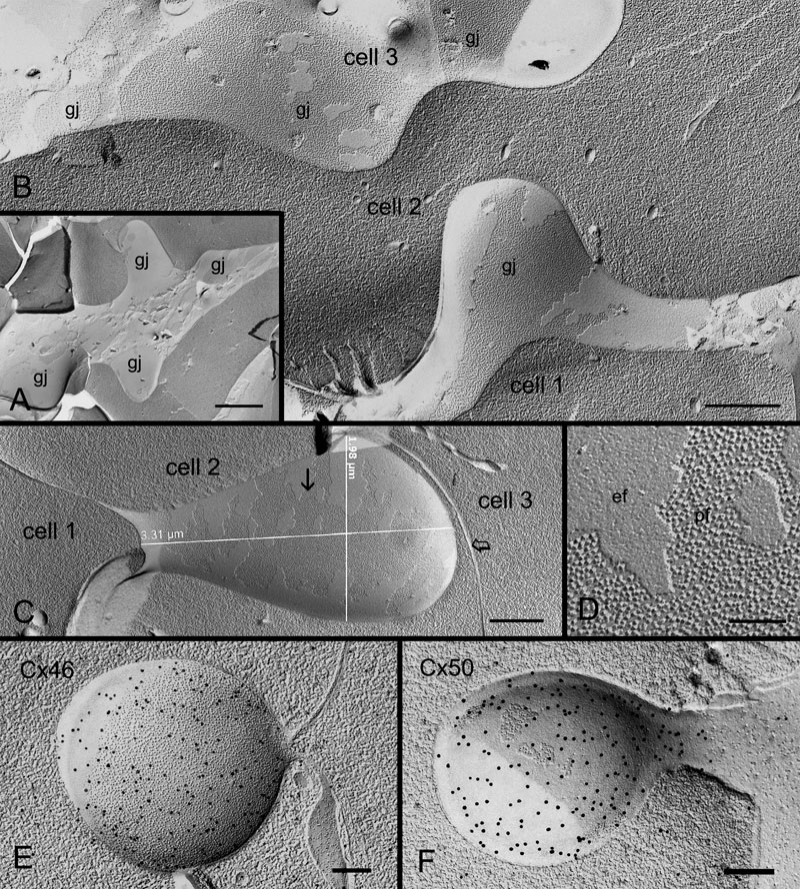
Freeze-fracture TEM and freeze-fracture immunogold labeling of Cx46 and Cx50 in ball-and-socket gap junctions of embryonic chicken lens fibers. **A**: Freeze-fracture TEM showing distribution of a cluster of ball-and-sockets containing gap junctions (gj). **B**: Higher magnification reveals the presence of gap junction plaque in the entire ball-and-socket domain (from cell 1) which protrudes into the cytoplasm of cell 2. The tip of this gap junction is in close proximity to the cell membrane of cell 3 which contains several flat gap junctions (gj). **C**: An elongated ball-and-socket gap junction, ~3.5 μm long and ~1.8 μm wide, from cell 1 protrudes deeply into the neighboring cell 2, and almost makes direct contact with the lateral cell membrane (open arrow) of cell 3. **D**: High magnification from the area marked by arrow in **C** showing gap junction particles (connexons) clearly seen on the P-face (pf) of the junctional membrane. **E** and **F**: FRIL shows the specific labeling of Cx46 and Cx50 antibodies in the ball-and-socket gap junctions, respectively. The scale bar indicates 1 μm in **A**; 500 nm in **B** and **C**; 100 nm in **D**, and 200 nm in **E** and **F**.

**Figure 5:**
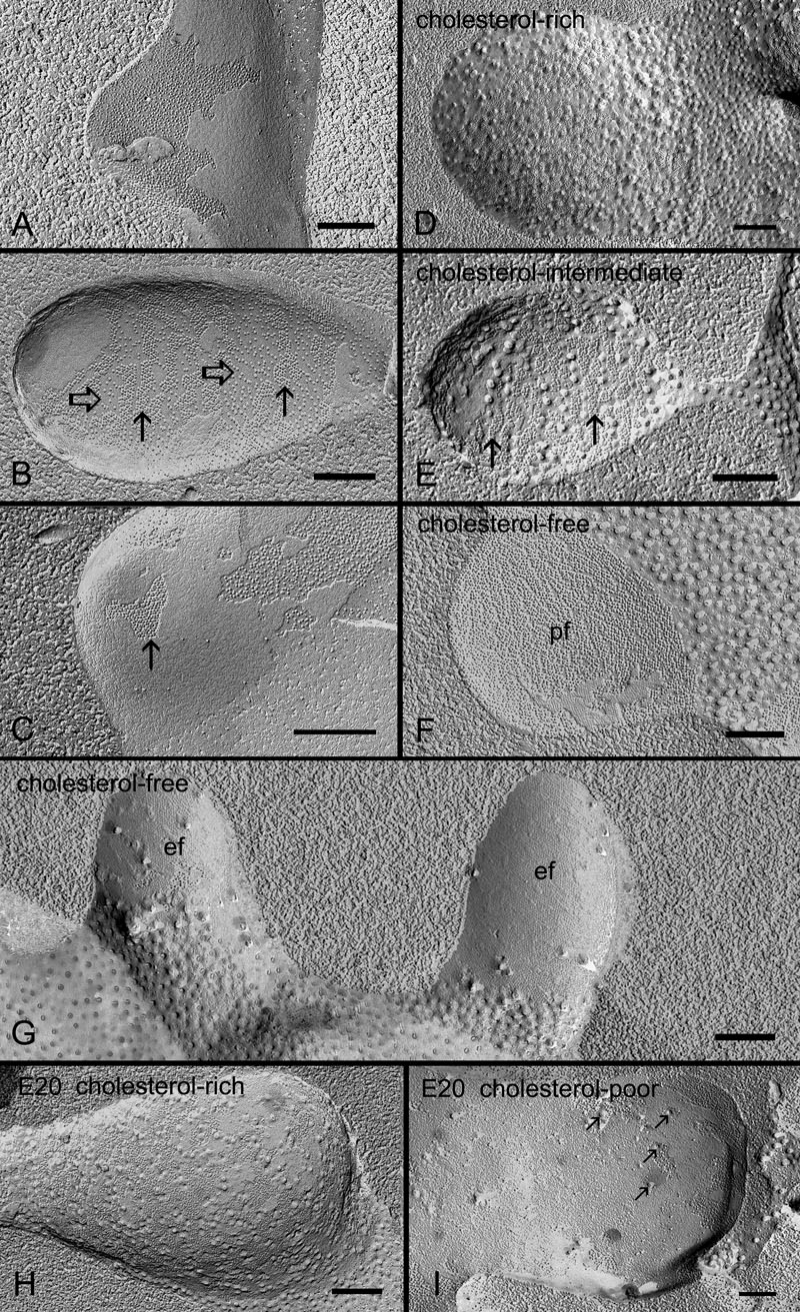
Freeze-fracture TEM and cholesterol distribution of ball-and-socket gap junctions in embryonic and adult chicken lens fibers. **A**: Ball-and-socket gap junction with loosely-packed configuration of connexons found in the outer cortex (0–400 μm from the surface). **B**: Ball-and-socket gap junction with a mixture of loosely-packed (open arrows) and crystalline-arranged (arrows) connexons found in the deeper region of the outer cortex. **C**: Ball-and-socket gap junction with crystalline-packed connexons (arrow) found in the inner cortex (400–800 μm). **D**: Cholesterol-rich ball-and-socket gap junction found in the outer cortex as determined by filipin cytochemistry in conjunction with freeze-fracture TEM. This gap junction exhibits loosely-packed connexons. **E**: Cholesterol-intermediate ball-and-socket gap junction found in the deeper region of the outer cortex. This gap junction displays distinct rows of crystalline-packed connexons (arrows) and few loose connexons. **F** and **G**: Cholesterol-free ball-and-socket gap junctions distributed in the inner cortex. These gap junctions contain the crystalline-packed configuration of connexons clearly seen on the P-face (pf) of the membrane in (**F**) and on the E-face (ef) of the membrane in (G). **H** and **I**: The presence of both cholesterol-rich and cholesterol-poor gap junctions in ball-and-sockets of the embryonic lens at E20. Several filipin-cholesterol-complexes (arrows) are indicated in the ball-and-socket in (**I**). The scale bars indicate 200 nm.

By taking advantage of our advanced freeze-fracture technique for routinely making large intact replicas [20,21], we were able to systematically examine the ball-and-sockets in the outer and inner cortices in both embryonic and adult lenses. To our surprise, from the total 142 ball-and-sockets examined, gap junctions were specifically associated with all ball-and-sockets examined (Figure 4 and Figure 5).

In the embryonic chicken lens, the ball-and-socket gap junctions were frequently found in clusters (Figure 4A). Many ball-and-socket gap junctions often protruded deeply into neighboring cells by as much as 3 μm in diameter (Figure 4B,C). The ball-and-socket gap junctions were confirmed to contain both Cx46 and Cx50 as judged by their specific labeling of both Cx46 and Cx50 antibodies in the ball-and-sockets with the freeze-fracture immunogold labeling technique (Figure 4E,F).

In the adult chicken lens, gap junctions were also associated with all ball-and-sockets examined in the outer and inner cortical fibers. These gap junctions exhibited three different packing configurations of connexons, i.e., loosely-packed (Figure 5A), mixture of loosely- and crystalline-packed (Figure 5B), and crystalline-packed (Figure 5C). The occurrence of these three different connexon arrangements in ball-and-socket gap junctions during fiber cell maturation is same as those previously reported for the flat fiber gap junctions in the outer and inner cortical fibers [21]. It should be noted that entire domains of almost all the ball-and-sockets examined in this study were fully occupied by gap junctions in both embryonic and adult chicken lenses (Figure 4 and Figure 5).

**Figure 6:**
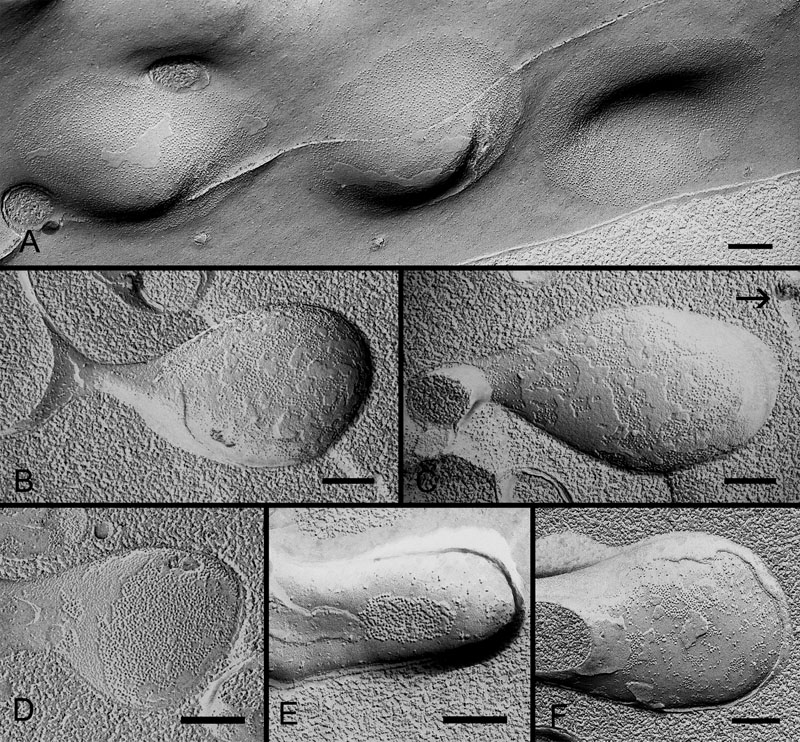
Freeze-fracture TEM of ball-and-socket gap junctions in monkey lens fibers. **A**: A cluster of three shallow ball-and-socket gap junctions are seen in superficial fibers of a young monkey (1.5 years old). **B** and **C**: Elongated ball-and-socket gap junctions with loosely-packed connexons in superficial cortical fibers of a mature monkey (20 years old). Arrow indicates the lateral cell membrane of adjacent cell. **D**, **E**, and **F**: Three different arrangements of connexons are associated with ball-and-sockets in the deeper mature cortical fibers: (**D**) A ball-and-socket completely occupied by connexons, (**E**) A ball-and-socket partially occupied by connexons, and (**F**) A ball-and-socket occupied by fragmentary gap junction plaques with disorganized connexons. Ball-and-socket gap junctions in **E** and **F** may be in a degradation stage. The scale bars indicate 200 nm.

In both young and adult monkey lenses, although all 103 ball-and-sockets examined were specifically associated with gap junctions, the configurations of these ball-and-socket gap junctions differed considerably in different cortical regions of the lens (Figure 6). While several newly-formed (Figure 6A) and complete (Figure 6B-D) ball-and-socket gap junctions were seen in the superficial differentiating fiber cells in both young (1.5 years old) and old (20 years old) lenses, some ball-and-sockets displayed only partial occupancy of connexons (Figure 6E) or disorganized connexons (Figure 6F) in the deeper cortical fibers, suggesting possible degradation of some connexons in the mature ball-and-socket gap junctions.

### Formation of gap junctions in ball-and-sockets in the embryonic chicken lens

**Figure 7:**
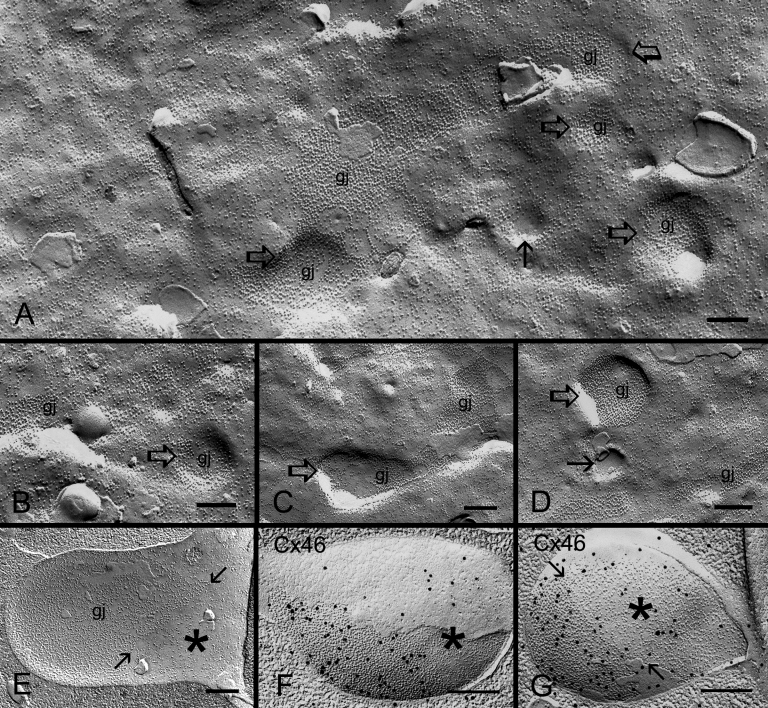
Formation of ball-and-socket gap junctions in embryonic chicken lens fibers. **A**: An overview of superficial cortical fiber cells during early-stage formation of ball-and-socket gap junctions (gj) as seen in small invaginations and concavities (open arrows). Some small concavities (arrows) are devoid of connexons. Others contain loosely scattered connexons which are directly associated with nearby pools of flat membrane connexons, suggesting that ball-and-socket connexons perhaps migrate from nearby existing gap junctions of the flat membrane. **B**, **C**, and **D**: A representative profile showing examples of early ball-and-socket gap junction formation. Note that differing amounts of connexons are found distributed inside the concavities (arrow and open arrows), suggesting that concavities are formed before the migration (or insertion) of connexons. **E**: A well formed ball-and-socket domain is almost completely occupied by gap junction connexons. The non-junctional portion is indicated by asterisk. **F** and **G**: FRIL shows some immunogold particles for specific labeling of Cx46 antibody are scattered in the non-junctional portion (asterisk) inside the ball-and-socket, suggesting the presence of individual connexons for completion of gap junction formation in this area. The scale bars indicate 200 nm.

It is important to find out how the ball-and-socket gap junctions are formed because these junctions are selectively associated with distinct membrane domains possibly for a particular function during fiber cell differentiation. Formations of gap junctions in ball-and-sockets were found more frequently in the superficial differentiating fiber cells. The initial indication for the formation of ball-and-socket gap junctions appeared to begin with the formation of small invaginations or concavities from the membrane surface (Figure 7A-D). Since the membrane particles (~9 nm) distributed within the small concavities were directly connected to the nearby newly-formed gap junctions on the flat membrane, these small concavities could therefore be considered as early ball-and-socket gap junctions (Figure 7A-D). Additionally, since these concavities contained varying numbers of connexons, ranging from zero to full occupancy, it is conceivable that the concavities were formed first followed by insertion or migration of connexons (Figure 7A-D). Also, some ball-and-sockets were not entirely occupied by gap junctions (Figure 7E-G), suggesting that the formation of a complete gap junction plaque was still underway. FRIL studies confirmed that some immunogold particles for the Cx46 antibody labeling were scattered in the non-junctional area of the ball-and-sockets (Figure 7F-G), indicating the presence of some individual connexons in this area for continued gap junction formation.

### Different cholesterol distributions in ball-and-socket gap junctions in chicken lens fibers

Filipin cytochemistry revealed that the cholesterol-rich, cholesterol-intermediate and cholesterol-free subtypes of ball-and-socket gap junctions were found in cortical fibers of the embryonic and adult chicken lenses (Figure 5D-I) as those previously reported for the flat fiber gap junctions seen in the embryonic and adult chicken lenses [20,21].

### Structure, distribution and high cholesterol content of protrusions in chicken lens fibers

**Figure 8:**
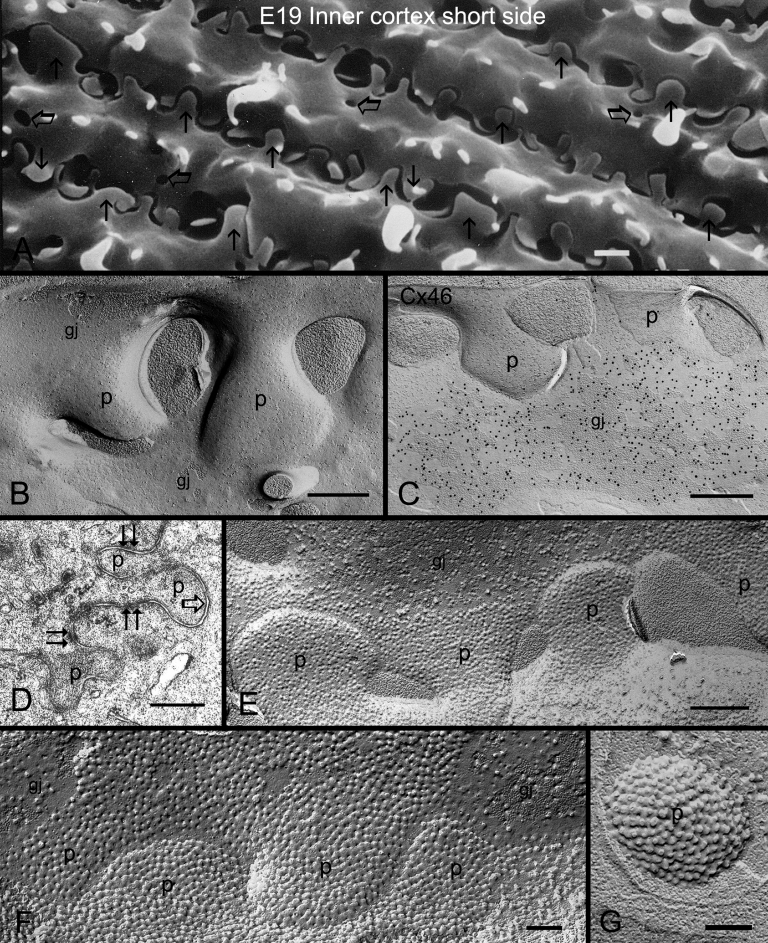
Structure and cholesterol distribution of protrusions in mature cortical fibers of the embryonic chicken lens fibers. **A**: SEM showing the distribution of numerous protrusions (arrows) from the corners of fiber cells in the inner cortex. Several small sockets (open arrows) of ball-and-sockets are also seen in the inner cortex. Note that two different sizes of protrusions from adjacent cells are often paired together for interlocking. **B**: Freeze-fracture TEM showing the absence of any gap junction on protrusions (p), although two gap junctions (gj) are found in close proximity to the protrusions (p). **C**: Freeze-fracture immunogold labeling confirms the absence of labeling for the Cx46 antibody in the protrusions (p). Instead, Cx46 antibody specifically labels the nearby large gap junction (gj). **D**: Thin-section TEM reveals the complex configuration of protrusions (p) without the association of gap junction (open arrow) with the protrusions. Surprisingly, several adherens junctions (paired arrows) are found associated with the neck portion of these protrusions. **E**: Filipin cytochemistry in conjunction with freeze-fracture TEM shows that a cluster of the protrusions (p) contain consistently high amounts of cholesterol (filipin-cholesterol-complexes, FCCs). **F**: At higher magnification, the protrusions display a high density of membrane cholesterol (i.e., 402 FCCs/μm^2^ membrane). Note that the adjacent gap junction (gj), classified as the cholesterol-rich subtype, contains only one half of FCCs (i.e., 188 FCCs/μm^2^ membrane) distributed in the protrusions. **G**: A top-viewed protrusion (p) in the cytoplasm showing a high density distribution of filipin-cholesterol-complexes. The scale bar indicates 1 μm in **A**, 500 nm in **B**, **C**, **D**, **E**, and 200 nm in **F**, and **G**.

Protrusions were the most prominent interlocking device (interdigitation) between adjacent lens fibers in all species studied. They were found distributed primarily along the corners of hexagonal fiber cells with high frequency in inner cortex in the embryonic chicken lens (Figure 8A,B). Structurally, protrusions were generally composed of the head and neck portions which enabled them to interlock tightly with those of the neighboring fibers (Figure 8A,B). Freeze-fracture TEM revealed that some gap junctions were often found distributed in close proximity, but the protrusions were not directly associated with any gap junctions (Figure 8B). The absence of gap junctions in protrusions was further confirmed by freeze-fracture immunogold labeling for Cx46 antibody for which positive labeling was only observed in the nearby gap junction (Figure 8C). The lack of gap junctions in the protrusions was also evidenced with thin-section TEM (Figure 8D). Instead, adherens junctions were often found associated with protrusions (Figure 8D). Finally, filipin cytochemistry in conjunction with freeze-fracture TEM demonstrated that protrusions consistently contained high amounts of cholesterol (FCCs; filipin-cholesterol-complexes) in both outer and inner cortical fibers during fiber differentiation and maturation (Figure 8E-G). It was estimated that the ratio for the amounts of cholesterol between the protrusions (i.e., 402 FCCs/μm^2^ membrane) and the cholesterol-rich gap junctions (i.e., 188 FCCs/μm^2^ membrane) seen in the ball-and-sockets was approximately 2:1. Protrusions showed similar structure, distribution and high cholesterol content in various cortical regions of the adult chicken lens (data not shown).

## Discussion

### Ball-and-sockets and protrusions are two distinct membrane domains in the lens

The lens organ possesses complex membrane interdigitations such as ball-and-sockets, protrusions, spikes, mounds, and tongue-and-grooves between fiber cells of various regions [14,31,33,44,45,47]. Structurally, the ball-and-sockets and protrusions resemble each other and have been suggested in many earlier studies to play important roles in maintaining structural stability between fiber cells during deformation of lens shape for visual accommodation [14,31-33,35]. However, it is not known why the lens requires many diverse interdigitations for maintaining normal transparent lens. The results of this study show, for the first time, that ball-and-sockets and protrusions are two distinct membrane domains for different functional roles during fiber cell differentiation and maturation in the lens. First, ball-and-sockets and protrusions exhibit different distributions, frequencies and specific structural features in different fiber regions of the lens. Ball-and-sockets are distributed on both long and short sides of hexagonal fiber cells in the superficial cortex and, with reduced frequency, in the deeper cortical regions. In contrast, protrusions are located mainly along the corners of hexagonal fiber cells in the outer cortex and, with increased frequency, in the deeper cortical regions of the lens. These differences have been regularly described in various species such as birds, rodents and primates [14,31-33,35,49]. At the ultrastructural level, the entire “ball” of a given cell protrudes into the invaginated “socket” inside the adjacent fiber cell to form a ball-and-socket interlocking domain. This unique configuration allows a given ball-and-socket to protrude deeply into the cytoplasmic core of the neighboring cell to increase cell membrane surface area for both individual fiber cells (Figure 2, Figure 4, Figure 5, Figure 6, and Figure 7). In contrast, protrusions along the corners of a given cell mainly make secure interdigitations (interlocking) with those of the adjacent cell, and they normally do not protrude deeply into the cytoplasmic core (Figure 8). Second, the most important difference between the ball-and-socket and protrusion is that gap junctions are present in all ball-and-sockets examined (i.e., 142 for chicken lenses and 103 for monkey lenses; Figure 2D,E, Figure 4, Figure 5, Figure 6, and Figure 7), but not in any protrusion (Figure 8). Third, ball-and-sockets and protrusions contain differing amounts of cholesterol. Filipin cytochemistry study reveals that cholesterol content is high in the protrusions, but is low or non-existent in the ball-and-sockets (Figure 5 and Figure 8), suggesting that the high cholesterol content would make the protrusion membrane less deformable [50,51] and thus would enhance the stability of these fiber-to-fiber interlocking devices.

### Functional importance of the ball-and-socket gap junctions in the lens

It is intriguing that a large number of gap junctions are distributed on the flat fiber cell membranes in the outer cortical region of the lens [2,6-8,13,18,52], but then why are many more gap junctions selectively associated with all ball-and-sockets found in the present study? The possible explanations are: first, ball-and-socket gap junctions are distributed primarily in the metabolically active fiber cells undergoing differentiation and growth. These young fiber cells possess all organelles required for cell differentiation and growth [53-55]. It is conceivable that the presence of numerous ball-and-socket gap junctions in these metabolically active cortical regions would significantly facilitate the movement of ions and metabolites required for fiber cell differentiation and growth. The possible facilitation for cell-to-cell communication by ball-and-socket gap junctions could be achieved through a significant expansion of their membrane surface areas into neighboring cells (Figure 4 and Figure 6). These ball-and-socket gap junctions would have a better spatial advantage for cell-to-cell communication than those gap junctions of the flat cell membranes. Second, the ball-and-socket gap junctions, like flat gap junctions, also undergo structural remodeling during fiber cell maturation and aging in the adult chicken lens [21]. These gap junctions display characteristic crystalline-packed connexons with cleavage of the COOH-terminus of Cx50 in the deep mature cortical fibers [21]. They represent a group of less active gap junctions associated with fiber cell maturation and aging [21]. In addition, this study reveals that in the superficial young cortical region almost all ball-and-socket domains are occupied by complete gap junction plaques (Figure 4 and Figure 6) whereas in the deeper mature cortical region the fragmentary gap junction plaques with disorganized connexons are usually associated with the domains (Figure 6). Thus, the decrease in ball-and-socket distribution together with the increase in degradation of ball-and-socket gap junctions in the deep mature fiber cells would further support the notion that ball-and-socket gap junctions are more functionally important for the metabolically active young fiber cells.

Additionally, the unique structural configuration of ball-and-socket gap junctions may also facilitate the internal circulation of ions in the lens as proposed by Mathias et al. [4,56-59]. The lens internal circulation model proposes that the inward current generated by the sodium ions enters the lens through extracellular spaces from both polar regions, and the sodium current eventually crosses the fiber cell membrane to enter the cell interior. The current within the cells subsequently flows outwardly from cell to cell through gap junctions to reach the equatorial epithelium and the sodium ions are pumped out by the Na/K ATPase at the lens equator. Thus, in this study, the large number of ball-and-socket gap junctions found near the equatorial region can effectively facilitate the outward current to flow toward the equatorial surface of the lens. Although the large number of ball-and-sockets is also present in the anterior and posterior portions of the differentiating young fibers (Figure 2 and Figure 3), most of their anterior and posterior ends actually do not reach the polar suture regions of the lens. Thus, the major portions of the newly differentiating fibers and their ball-and-socket gap junctions are mostly distributed near the equatorial region. For those ball-and-socket gap junctions found in the mature fibers of various regions, they frequently display only partial occupancy of junctional plaques with disorganized connexon arrangements, indicative of gap junction degradation (Figure 6). Thus, it is conceivable that a larger number of functioning ball-and-socket gap junctions distributed at the equatorial regions of the lens would play a role in the outward current of lens circulation.

### Functional importance of the protrusion interlocking devices in the lens

Protrusions are distributed along all corners of a given hexagonal fiber cell to form complex interlocking patterns with those of neighboring cells in various species examined [14,31-36,49]. These intricate patterns would be suitable for preventing the lateral sliding movements among fiber cells as proposed by several earlier studies [14,31-35]. Also, the frequent presence of adherens junctions near the protrusions (Figure 8) would further assist the protrusions in achieving this role. Furthermore, a consistent distribution of high concentrations of cholesterol in protrusions (Figure 8) would make the membrane less deformable and would thus be suitable for maintenance of fiber-to-fiber stability during lens shape change associated with visual accommodation.

**Figure 9:**
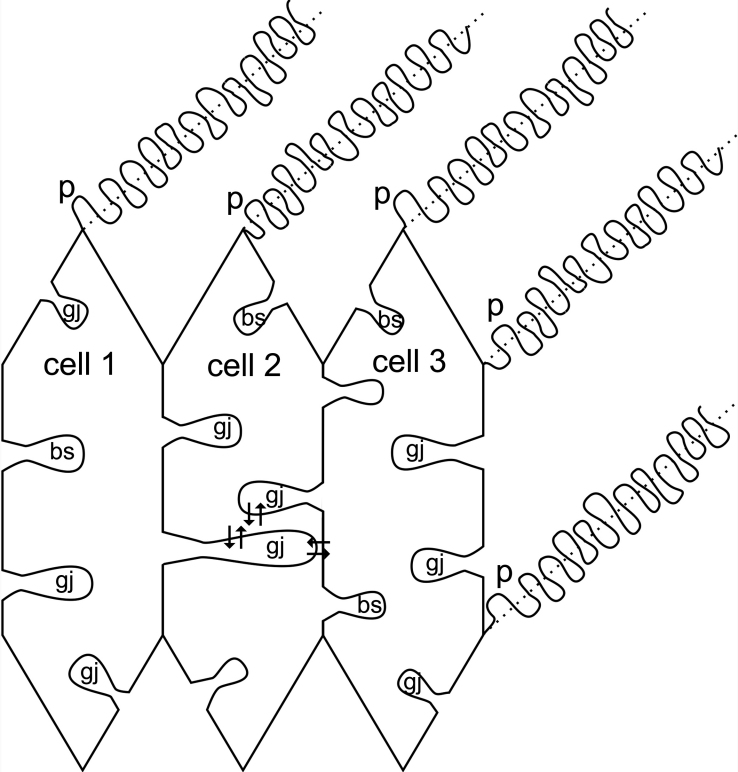
A summary diagram depicting the important structural and functional differences between the ball-and-socket (BS) and protrusions (P) in hexagonal cortical fiber cells. Ball-and-sockets are distributed on both the long and short sides of fiber cells and are more frequent in the superficial than in the deeper cortex. They are generally larger in size but smaller in number than the protrusions distributed primarily along the corners. Structurally, gap junctions (gj) are present in all ball-and-sockets examined, but not in protrusions. Many elongated ball-and-socket gap junctions protrude deeply into neighboring fiber cells. Also, while the ball-and-socket gap junctions contain significantly different amounts of cholesterol during fiber differentiation and maturation, all protrusions examined consistently contain high amounts of membrane cholesterol. The cholesterol ratio between protrusions and the cholesterol-rich gap junctions seen in ball-and-sockets is approximately 2:1. It is suggested that the unique structural configuration of ball-and-socket gap junctions may significantly facilitate cell-to-cell communication (arrows) between metabolically active young fiber cells in the superficial cortex. Also, the large number of ball-and-socket gap junctions found near the equatorial region may effectively facilitate the flow of outward current toward the equatorial surface for internal circulation of ions in the lens. The presence of high cholesterol content in protrusions would make these domain membranes less deformable, and would be more suitable for maintenance of fiber-to-fiber stability during visual accommodation.

In conclusion, the present study demonstrates that the ball-and-sockets and protrusions are two structurally and functionally distinct membrane domains in the lens (Figure 9). Ball-and-sockets are distributed on the long and short sides of fiber cells and are more frequent in the superficial cortex than in the deeper cortex. In contrast, protrusions are found regularly along the corners of hexagonal fiber cells in the outer cortex and, with increased frequency, in the deeper cortical regions of the lens. Importantly, gap junctions are selectively associated with all ball-and-sockets examined but not with any protrusion. It is suggested that the unique structural configurations of ball-and-socket gap junctions may significantly facilitate their communicating role between metabolically active young fiber cells by protruding deeply into neighboring cells to increase membrane surface areas for cell-to-cell communication. Also, the large number of ball-and-socket gap junctions found near the equatorial region may effectively facilitate the outward current to flow toward the equatorial surface for internal circulation of ions in the lens. In contrast, the presence of consistently high cholesterol content in protrusions would make these interlocking domain membranes less deformable, and would be more suitable for maintaining the fiber-to-fiber stability.
